# Supplementary material for: The effects of genital myiasis on the diversity of the vaginal microbiota in female Bactrian camels
Source: BMC Vet Res. 2022 Mar 5;18:87. doi: 10.1186/s12917-022-03189-5 (PMC8897907; doi:10.1186/s12917-022-03189-5)
Supplement: Supplementary file 5 — Additional file 5. [file 12917_2022_3189_MOESM5_ESM.zip › MPL201709200_16s_yy/Treat1/css/js_css/jsPDF-master.zip!/jsPDF-master/index.html]

jsPDF - Create PDFs with HTML5 JavaScript Library


Star me on GitHub

Tweet
Follow @MrRio

Download Now

Version 0.9.0rc2

# jsPDF

#### HTML5 JavaScript PDF generation library from @MrRio at Parallax

## Have a play.

A HTML5 client-side solution for generating PDFs. Perfect for event tickets, reports, certificates, you name it!

**No servers were used in the making of this demo.**

Choose example

Hold your horses...

Download PDF

 Auto refresh on changes?
Run Code

Auto refresh disabled for this

## The Basics

Simply include the jsPDF library in your <head>, generate your PDF using the *many* built-in functions, then hook up a button to trigger the download. All the examples here use jQuery.

## Browser Compatibility

jsPDF will work in IE6+\*, Firefox 3+, Chrome, Safari 3+, Opera. For IE9 and below, we lazily load a Flash shim called Downloadify which enables the files to be downloaded.

\* Current build does not have IE6-9 shim enabled

**NB:** UTF-8 is *not* supported by default, however there might be some plugins allowing you to use it, such as *addHTML*

十五向學,三十而立,四十而不惑,五十而知天命,六十而耳順,七十而從心欲，不踰矩.

## Need Help?

Send me a tweet and I'll see if I can help :)

## Credits

Big thanks to Daniel Dotsenko from Willow Systems Corporation for making huge contributions to the codebase.

Thanks to Ajaxian.com for featuring us back in 2009.

Everyone else that's contributed patches or bug reports. You rock.

## License

(MIT License)

Copyright (c) 2010-2016 James Hall, https://github.com/MrRio/jsPDF

Permission is hereby granted, free of charge, to any person obtaining
a copy of this software and associated documentation files (the
"Software"), to deal in the Software without restriction, including
without limitation the rights to use, copy, modify, merge, publish,
distribute, sublicense, and/or sell copies of the Software, and to
permit persons to whom the Software is furnished to do so, subject to
the following conditions:

The above copyright notice and this permission notice shall be
included in all copies or substantial portions of the Software.

THE SOFTWARE IS PROVIDED "AS IS", WITHOUT WARRANTY OF ANY KIND,
EXPRESS OR IMPLIED, INCLUDING BUT NOT LIMITED TO THE WARRANTIES OF
MERCHANTABILITY, FITNESS FOR A PARTICULAR PURPOSE AND
NONINFRINGEMENT. IN NO EVENT SHALL THE AUTHORS OR COPYRIGHT HOLDERS BE
LIABLE FOR ANY CLAIM, DAMAGES OR OTHER LIABILITY, WHETHER IN AN ACTION
OF CONTRACT, TORT OR OTHERWISE, ARISING FROM, OUT OF OR IN CONNECTION
WITH THE SOFTWARE OR THE USE OR OTHER DEALINGS IN THE SOFTWARE.

© 2016 James Hall - Privacy Policy
